# Liquid Carbon-Boron Cluster Additives for Mixed Amine-50

**DOI:** 10.3390/molecules30092037

**Published:** 2025-05-03

**Authors:** Xiaogang Mu, Shenghui Wang, Fanzhi Yang, Hao Li

**Affiliations:** 1 Zhijian Laboratory, Rocket Force University of Engineering, Xi’an 710025, China; 2 Beijing Institute of Technology, Advanced Research Institute of Multidisciplinary Science, Beijing 100081, China

**Keywords:** liquid propellant, energy density, carborane, cluster, theoretical specific impulse

## Abstract

Boron-containing compounds, known for their high calorific value, can significantly enhance the energy density of traditional liquid propellants. Through precise chemical modification of the C-H bonds in *ortho*-carborane, a novel liquid cluster material was synthesized: C,C′-Bis(2-ethylhexanoylmethyl)-*o*-carborane (**BEHMC**). This compound exhibits excellent stability, density, and energy properties. By combining **BEHMC** with mixed amine-50, a cluster liquid propellant was formulated, and its key performance metrics such as density, freezing point, mass calorific value, and volumetric calorific value were evaluated. The propellant achieved a density of 0.884 g·cm^−3^, a freezing point below −55 °C, a mass calorific value of 41.13 kJ·g^−1^, and a volumetric calorific value of 36.36 kJ·cm^−3^. The theoretical specific impulse reached 346 s, and the theoretical density impulse was 2.99 × 10^6^ N·s·m^−3^. Compared to the traditional mixed amine-50/red fuming nitric acid propellant, the cluster liquid propellant developed in this study maintains a similar calorific value while improving the theoretical specific impulse and density impulse by 2.3% and 3.8%, respectively.

## 1. Introduction

Liquid propellants are the core of spacecraft propulsion systems and are categorized into cryogenic and storable types. Cryogenic propellants, such as kerosene–liquid oxygen and liquid hydrogen–liquid oxygen combinations, face challenges in storage and transportation due to low-temperature requirements, necessitating expensive cryogenic containers and precise temperature control [1,2,3]. Additionally, ignition is difficult, requiring complex ignition systems that may fail and affect missions [4,5,6,7,8,9]. In contrast, hypergolic propellants can be stored stably at room temperature, simplifying ground equipment requirements and reducing operational costs [10,11,12]. They provide strong thrust, making them suitable for long-distance missions. More importantly, hypergolic propellants can ignite upon contact, eliminating the need for additional ignition devices, simplifying engine design, and enhancing safety [13,14,15]. Their rapid start-up and repeat ignition capabilities are crucial for missions requiring multiple orbital adjustments [16,17,18]. Therefore, hypergolic propellants have been widely used in China’s large launch vehicles [19,20,21].

Energy density, which refers to the amount of energy stored per unit volume or mass of a substance, is one of the key indicators for evaluating fuel performance. With the rapid development of beyond-visual-range and non-contact warfare, the demand for higher energy density in propellants is increasing. Although hypergolic fuels exhibit excellent combustion and propulsion performance, their low density and energy levels make it difficult to meet the requirements of modern high-payload, high-specific-impulse propulsion systems [22,23]. Therefore, developing a high-energy-density fuel that is easy to maintain and use at room temperature is crucial for achieving high-speed, high-maneuverability, and long-endurance propulsion systems, which is significant for the development of high-performance propellants.

Currently, enhancing energy density through the chemical synthesis of new fuel molecular structures is challenging. Since the 1990s, research led by NASA and other countries has explored a new method to improve liquid fuel energy density: adding metal particles to fuels. This research direction involves both theoretical exploration and practical engine testing [24]. However, the addition of metal particles often leads to poor compatibility stability and particle sedimentation. It has been reported that boron-based compounds, known for their good compatibility and high calorific value, have been applied to enhance propellant performance [25,26,27]. Among them, carborane derivatives are typical performance modifiers. Carboranes are a class of closed-cage boron compounds, and their derivatives exhibit significant volumetric calorific values (above 5.73 × 10^4^ kJ·L^−1^) and high densities (0.95 g·cm^−3^). Their unique electron-deficient structures are highly compatible with oxidizing substances, and they release a large number of electron-deficient fragments during combustion, significantly improving fuel energy density [28,29,30]. Based on this, our research group has conducted studies on applying carborane derivatives to hypergolic propellants. In this study, a liquid cluster material, C,C’-Bis(2-ethylhexanoylmethyl)-*o*-carborane, was synthesized based on *ortho*-carborane and applied to enhance the energy density and specific impulse of mixed amine-50 fuel.

Wang et al. first discovered the all-boron fullerene B40 (borospherene) in 2014 through gas-phase photoelectron spectroscopy and quantum chemical calculations [31]. The unique D2d-symmetric cage structure, distinct from carbon fullerenes like C60, consists of interwoven boron chains with delocalized three-center σ and π bonds, exhibiting three-dimensional aromaticity. While the liquid-phase application of boron cages (e.g., B40) remains challenging due to their solid-state synthesis requirements and stability limitations, their high surface area and electron-deficient nature inspire the design of energetic materials. The *ortho*-carborane derivatives in this study enhance liquid system stability and energy density through chemical modifications, synergistically demonstrating the potential of boron multi-center bonding with borospherene studies. Future work may explore boron cage derivatives as solid additives to further improve propellant performance.

## 2. Experimental

### 2.1. Reagents and Instruments

The reagents used in this study included mixed amine-50, which was prepared in-house in the laboratory [32], *ortho*-carborane (analytical grade), (Shanghai Bide Pharmaceutical Technology Co., Ltd., Shanghai, China). Paraformaldehyde (analytical grade), 2-ethylhexanoyl chloride (analytical grade), n-butyllithium (2.4 mol/L, analytical grade), triethylamine (analytical grade), and 2,4-dimethylaniline (analytical grade), (Shanghai Macklin Biochemical Technology Co., Ltd., Shanghai, China). Super-dry tetrahydrofuran (analytical grade) and super-dry dichloromethane (analytical grade), (Beijing Innochem Technology Co., Ltd., Beijing, China), while dichloromethane (analytical grade), n-hexane (analytical grade), and anhydrous sodium sulfate (analytical grade), (Beijing Tongguang Fine Chemicals Company, Beijing, China). The instruments employed in this research comprised a Bruker 400 MHz liquid nuclear magnetic resonance spectrometer and a Bruker VERTEX 80V Fourier-transform infrared spectrometer, both from Bremen, Germany. Thermal analysis was conducted using a Mettler Toledo TGA-1 thermogravimetric analyzer (Columbus, OH, USA). Density measurements were performed with a DMA4101 automatic densitometer (Anton Paar, Graz, Austria), and freezing points were determined using an ST-1568A freezing point analyzer (Beijing Xuxin Instrument Equipment Co., Ltd., Zhangjiakou, China). Calorimetric analysis was carried out with a PARR 6200 automatic oxygen bomb calorimeter (Moline, IL, USA), and rheological properties were assessed using a RHEOTEST laboratory viscometer rheometer (Ottendorf-Okrilla, Germany).

### 2.2. Synthesis and Characterization

#### 2.2.1. Synthesis of C,C′-Bis(2-ethylhexanoylmethyl)-*o*-carborane

**Figure 1:**
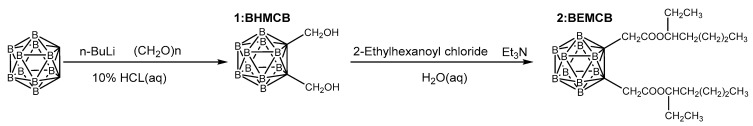
Synthesis route of C,C′-Bis(2-ethylhexanoylmethyl)-o-carboran.

(1) Bis(hydroxymethyl)-*o*-carborane (**1**, **BHMCB**)
  A solution of 5 g (34.67 mmol) of *ortho*-carborane (OCB) in 60 mL of super-dry tetrahydrofuran was prepared. Under nitrogen protection and at −78 °C, 30 mL of n-butyllithium (2.4 mol/L) was slowly added dropwise to the carborane solution, followed by stirring for 2 h. Subsequently, 2.19 g (72.81 mmol) of paraformaldehyde was added to the mixture in one portion at room temperature, and the reaction system was stirred for an additional 10 h. The reaction was quenched using a 10% HCl aqueous solution. After evaporating the solvent and water using a rotary evaporator, the residue was extracted with diethyl ether. The organic layer was washed three times with deionized water and dried over anhydrous sodium sulfate. After removing the solvent, the residue was recrystallized using a mixture of dichloromethane and n-hexane (Vdichloromethane/Vn-hexane, 1:5). The crystallized product was washed extensively with n-hexane to yield a white solid with a yield of 87%. Chemical shifts attributed for **BHMCB**: ^1^H NMR (400 MHz, CDCl3) δ (ppm): 4.23 (4H, d), 2.87 (2H, s), 1.39–3.48, (10H, br m); ^13^C NMR (101 MHz, CDCl3) δ (ppm): 78.18, 64.48; ^11^B (128 MHz, CDCl3) δ (ppm): −3.28 (d, *J* = 149.2 Hz), −11.15 (dd, *J* = 165.2, 89.9 Hz).

(2) C,C’-Bis(2-ethylhexanoylmethyl)-*o*-carborane (**2**, **BEMCB**):
  A mixture of 5 g (24.48 mmol) of **BHMCB** and 5.59 mL (56.30 mmol) of triethylamine was dissolved in super-dry dichloromethane. Under nitrogen protection and at −5 °C, 8.9 mL (53.85 mmol) of 2-ethylhexanoyl chloride was added dropwise to the solution. After the addition was complete, the reaction was allowed to warm to room temperature and stirred for 5 h. The reaction was then quenched with deionized water. The solvent and water were evaporated using a rotary evaporator, and the residue was extracted with diethyl ether. The organic layer was washed three times with deionized water and dried over anhydrous sodium sulfate. After removing the solvent, the residue was purified by column chromatography using a mixture of dichloromethane and n-hexane (1:10) to yield a pale yellow liquid with a yield of 80%. The synthetic route is shown in Figure 1.Chemical shifts attributed for **BEMCB**: ^1^H NMR (400 MHz, Chloroform-d) δ (ppm): 4.66 (q, *J* = 7.1, 6.2 Hz, 4H), 2.32 (tt, *J* = 13.2, 5.7 Hz, 2H), 1.55 (dddd, *J* = 53.5, 31.7, 13.4, 6.4 Hz, 10H), 1.32–1.23 (m, 4H), 0.91–0.85 (m, 12H). 1.39–3.48, (10H, br m); ^13^C NMR (101 MHz, CDCl3) δ (ppm): 174.45, 75.87, 62.42, 46.93, 31.25, 29.46, 25.02, 22.53, 13.83, 11.71; ^11^B (128 MHz, CDCl3) δ (ppm): −3.03 (d, *J* = 149.7 Hz), −10.29 (d, *J* = 162.4 Hz).

#### 2.2.2. Preparation of Cluster Liquid Propellant

Under continuous stirring, bis(hydroxymethyl)-*o*-carborane and C,C′-bis(2-ethylhexanoylmethyl)-*o*-carborane were added to the mixed amine-50 fuel in batches. The mixture was stirred for approximately 5 min to ensure complete dissolution and homogeneity, resulting in the formation of a uniform and transparent composite liquid propellant fuel.

#### 2.2.3. Characterization Methods and Testing Conditions

The characterization and testing of the composite fuel were conducted using a variety of methods under specific conditions. Fourier transform infrared spectroscopy (FTIR) analysis was performed with a KBr beam splitter, a resolution of 4 cm^−1^, 32 scans, and a scanning range of 400–4000 cm^−1^; for freezing point determination, a 20 mL sample of the composite fuel was placed in a dedicated container of the freezing point analyzer and tested at −60 °C; density measurements were carried out using an automatic densitometer, where a 5 mL sample of the composite fuel was tested at 20 °C, with the density value calculated as the average of three measurements; the combustion calorific value of the composite fuel was evaluated using an oxygen bomb calorimeter, with 0.3 g of the sample weighed for each test and an oxygen pressure maintained at 3 MPa; the kinematic viscosity of the two composite fuels was measured at 20 °C using a rotational rheometer; thermogravimetric analysis (TGA) was conducted under two sets of conditions: for the filler material, the temperature range was 30–350 °C, with a heating rate of 20 °C·min^−1^, a gas flow rate of 30 mL·min^−1^, and high-purity argon as the inert protective gas, while for the composite fuel, the temperature range was extended to 30–450 °C, maintaining the same heating rate, gas flow rate, and protective gas. These comprehensive tests provided detailed insights into the physical and chemical properties of the composite fuel.

## 3. Results and Discussion

### 3.1. Characterization and Analysis

#### 3.1.1. Structural Characterization of C,C′-Bis(2-ethylhexanoylmethyl)-*o*-carborane (**BEMCB**)

**Figure 2:**
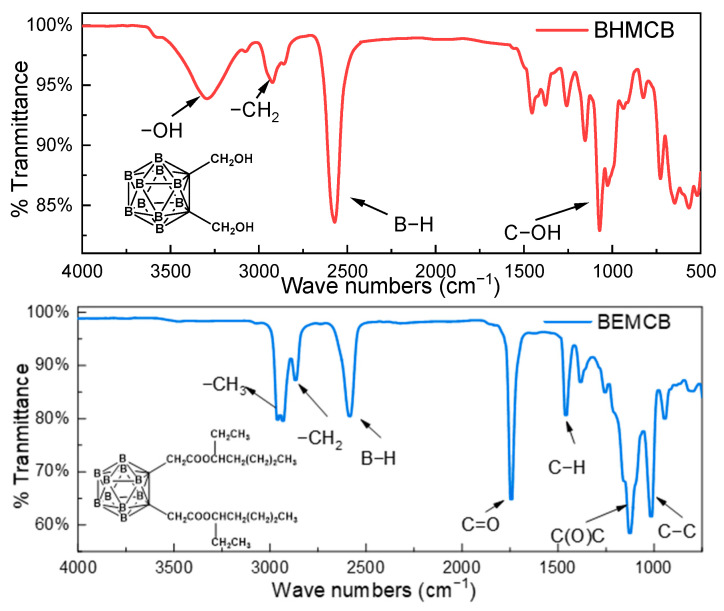
FTIR spectra of **BHMCB** and **BEMCB**.

Figure 2 presents the infrared (IR) spectrum of **BEMCB**, with the IR spectrum of **BHMCB** included for comparative analysis. The reaction conducted in this experiment was an esterification reaction, and the key criterion for determining its success lies in the disappearance of hydroxyl groups and the appearance of ester groups. From the IR spectra, it is evident that the B-H stretching vibration peak of **BHMCB** remains intact after the reaction, indicating that the B-H bonds in **BHMCB** were neither broken nor altered during the process. However, the hydroxyl (-OH) stretching vibration peak, originally located at 3293 cm^−1^, disappears in the spectrum of **BEMCB**. The -OH stretching vibration typically appears in the range of 3200–3600 cm^−1^, and its disappearance suggests that the hydroxyl groups in the reactants have participated in the chemical reaction and no longer exist as free hydroxyl groups, which is a significant indicator of successful esterification.

Simultaneously, compared to the spectrum of **BHMCB**, two new characteristic absorption peaks appear in the spectrum of **BEMCB**. One is the C=O stretching vibration peak at 1774 cm^−1^, which typically appears in the range of 1700–1800 cm^−1^ for ester groups, indicating the formation of a new C=O bond during the reaction, a hallmark of ester group formation. The other new absorption peak is the C(O)C stretching vibration peak at 1077 cm^−1^, which generally appears in the range of 1000–1300 cm^−1^. The presence of this peak further confirms the formation of the ester group, as the ester structure inherently contains a C(O)C bond fragment. By comparing the IR spectra of **BHMCB** and **BEMCB**, it can be preliminarily concluded that the structure of **BEMCB** aligns with expectations, and the esterification reaction successfully converted hydroxyl groups into ester groups, yielding the target compound **BEMCB**.

**Figure 3:**
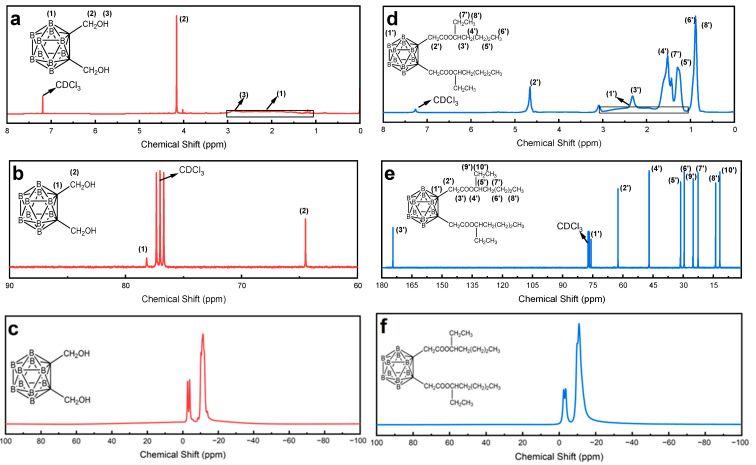
^1^H-NMR, ^13^C-NMR, and ^11^B-NMR spectra of **BHMCB** (**a**–**c**) and **BEMCB** (**d**–**f**), respectively.

Figure 3 presents the ^1^H NMR spectrum of **BEMCB**, with the spectrum of **BHMCB** provided for comparative analysis. The target product is a carborane ester compound, and the most compelling evidence for its formation is the observation of absorption peaks corresponding to methyl (-CH_3_), methylene (-CH_2_-), methine (-CH-), and boron-hydrogen (B-H) bonds, as well as the disappearance of the hydroxyl (-OH) peak. From the NMR spectrum, it can be observed that the -OH peak of **BHMCB**, originally located at 2.87 ppm, has disappeared, indicating that the hydroxyl groups in the reactants have participated in the chemical reaction and no longer exist as free hydroxyl groups. This is a significant indicator of successful esterification. Meanwhile, the absorption peaks of B-H bonds in the range of 1.39–2.91 ppm remain intact, demonstrating that the B-H bonds in **BHMCB** were neither broken nor altered during the reaction, preserving the original chemical structure.

Comparative analysis of the ^1^H NMR spectra between precursor **BHMCB** and target compound **BEMCB** systematically validates the structural integrity of **BEMCB**. The complete disappearance of hydroxyl-associated protons and the emergence of ester-specific resonances (e.g., carbonyl groups) unambiguously confirm the success of the esterification reaction. The anisotropic shielding effects induced by the carborane cage manifest as characteristic upfield shifts (δ 1.39–3.48 ppm) in boron-proximal protons, consistent with established electronic interactions in carborane-containing systems. Integration ratios for distinct proton environments (e.g., terminal alkyl chains vs. ester-linked groups) quantitatively align with theoretical predictions, while splitting patterns adhere to vicinal coupling relationships.

^13^C NMR analysis reveals significant electronic reorganization driven by esterification. For **BHMCB** (Figure 3b), signals at δ 78.18 and 64.48 ppm correspond to the hydroxyl-bearing carbon (-CH_2_-OH) and its adjacent carbon, respectively. In **BEMCB** (Figure 3e), the carbonyl carbon of the ester group (-COO-) resonates at δ 174.45 ppm, while the ether-linked carbon appears at δ 75.87 ppm. Methylene carbons adjacent to the ester oxygen (-OCOOCH_2_-) and other functional groups are observed at δ 62.42 and 46.93 ppm. Signals in the range δ 31.25–11.71 ppm correspond to alkyl carbons (-CH_2_- and -CH_3_) within the aliphatic chain, consistent with **BEMCB**’s structural framework. The emergence of the carbonyl carbon signal (Δδ ~30 ppm upfield shift for carborane-proximal carbons) directly confirms covalent bond transformation, with electron-withdrawing conjugation between the ester group and carborane cage inducing deshielding effects. These observations, combined with the hydroxyl-to-ester conversion in ^1^H NMR, collectively validate **BEMCB**’s structural fidelity. ^11^B NMR spectral evolution further corroborates symmetry-breaking dynamics. The transition from a double doublet (**BHMCB**) to a distinct doublet (**BEMCB**) confirms substituent-induced electric field gradient alterations in the carborane cage. Quadrupolar interactions (^11^B, I = 3/2) modulate peak multiplicity, where J-coupling variations (Δ*J* = +72.5 Hz) reflect structural distortion from icosahedral (Ih) to Cs symmetry. Boron deshielding (Δδ = +0.86 ppm, δ −11.15 → −10.29 ppm) arises from electron-withdrawing conjugation between the ester group and carborane cage, aligning with anisotropic shielding patterns observed in ^1^H/^13^C NMR. These spectral changes validate B-H through-space coupling attenuation and dipole moment redistribution induced by substituent effects.

#### 3.1.2. Thermal Stability Analysis

**Figure 4:**
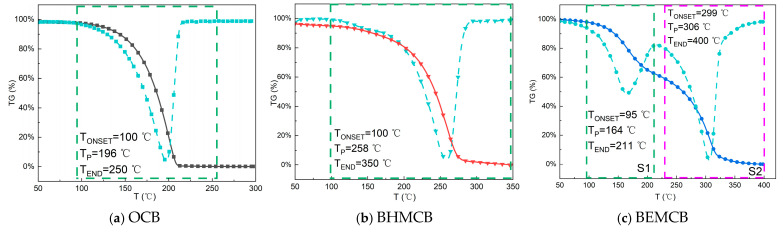
Thermo-gravimetric curves of OCB (**a**), **BHMCB** (**b**) and **BEMCB** (**c**). (Curves in black, red, and blue represent the thermogravimetric (TG) profiles of OCB, **BHMCB**, and **BEMCB**, respectively. Cyan curves correspond to their derivative thermogravimetric (DTG) profiles. Dashed boxes, color-coded by the thermal decomposition stage, delineate temperature ranges for distinct degradation processes.

To evaluate the thermal stability of OCB, **BHMCB**, and **BEMCB**, TGA measurements were conducted on these three samples. The heating rate during the test was set at 20 °C/min, with a temperature range of 30–350 °C. Figure 4 presents the thermogravimetric curves of these three samples.

From Figure 4a,b, it can be observed that the initial mass of all samples at the beginning of the analysis was close to 100%, indicating no significant mass loss in the initial stage. The TGA results show that OCB experienced significant mass loss between approximately 150–250 °C, with a sharp decline in this temperature range indicating its main thermal decomposition process. In contrast, **BHMCB** exhibited noticeable mass loss between approximately 100–350 °C, but its thermal decomposition rate was slower, demonstrating a broader thermal decomposition temperature range. In terms of thermal stability, OCB showed lower stability, almost completely decomposing at around 250 °C. On the other hand, **BHMCB** exhibited higher thermal stability, with its decomposition process continuing until approximately 350 °C, indicating that it could maintain stability for a longer duration at high temperatures. Regarding residual mass, OCB showed almost no residual mass at around 250 °C, indicating complete decomposition at this temperature. In contrast, **BHMCB** still retained some residual mass at the same temperature, suggesting incomplete decomposition. The results in Figure 4c reveal that the thermal decomposition of **BEMCB** occurred in two stages. The first stage, from 95–211 °C, showed a gradual decline in mass. The second stage, from approximately 299–400 °C, exhibited the maximum rate of mass loss. Compared to OCB and **BHMCB**, **BHMCB** completely decomposed at 400 °C, indicating superior thermal stability over the other two. Additionally, according to the DTG analysis results, OCB reached its maximum decomposition rate at 196 °C, while **BHMCB** and **BEMCB** reached their maximum thermal decomposition rates at 258 °C and 306 °C, respectively, further confirming that **BEMCB** has the best thermal stability.

In summary, the TG-DTG analysis provided a detailed assessment of the thermal stability of OCB, **BHMCB**, and **BEMCB**. The results indicate that **BEMCB** exhibits better thermal stability at high temperatures, which may be attributed to its long-chain asymmetric structure. According to the thermal analysis results, the esterification reaction converts the hydroxyl groups of alcohols into ester bonds, which generally have higher bond energy than the O-H bonds in alcohols. This change significantly enhances the thermal stability of the molecule, as ester bonds are more resistant to breaking at high temperatures, thereby reducing the likelihood of molecular decomposition. Furthermore, the asymmetric long-chain structure of **BEMCB** provides a shielding effect, protecting functional groups such as ester groups from high-temperature effects. The bulky structure of the long-chain ester groups reduces unfavorable intermolecular interactions, enhancing the overall stability of the molecule. This shielding effect is akin to forming a protective layer around the molecule, reducing internal molecular vibrations and rotations at high temperatures, thereby lowering molecular activity. The long-chain asymmetric structure of the ester also results in strong intermolecular interactions, such as van der Waals forces, which more effectively restrict thermal motion at high temperatures, reducing molecular vibrations and rotations and decreasing molecular activity. Compared to **BHMCB**, a diol, the long-chain asymmetric structure of **BEMCB** reduces the number of reactive sites. This reduction not only decreases the reactivity of the molecule at high temperatures but also improves its overall thermal stability.

### 3.2. Performance Characterization and Analysis of Composite Fuels

#### 3.2.1. Compatibility and Stability Testing

As shown in Table 1, two types of composite fuels were prepared: Composite Fuel B (**BHMCB**/mixed amine-50) and Composite Fuel C (**BEMCB**/mixed amine-50). The base fuel A (mixed amine-50) was used as the blank group.

**Figure 5:**
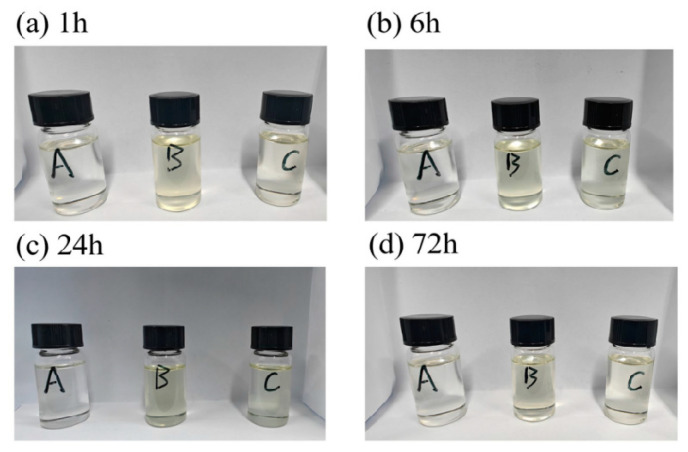
Compatibility of **BHMCB** and **BEMCB** in Mixed Amine-50.

To verify the compatibility of **BHMCB** and **BEMCB** with liquid propellant fuels, a compatibility test was designed. The overall plan of the test is to compare and analyze the compatibility and stability of different types of boron-based fillers in liquid fuels by setting up a blank control group and conducting experiments under the same conditions. The specific test procedure involves mixing each group of samples under ultrasonic conditions for 15 min, followed by allowing them to stand. Observations and photographic records are then taken for the three groups of samples after standing for 1 h, 6 h, 24 h, and 72 h. The stability of the blended fuel during long-term storage is observed to validate the compatibility of the fillers with the fuel. Figure 5 shows the condition of each group of samples after standing for different periods of time.

Based on the experimental results, it was observed that the compounded fuel did not show any significant changes in appearance and properties after long-term static storage. No precipitate formation was observed, nor was there any bubble generation, and there was no noticeable pressure upon opening. The compatibility test results confirmed that **BHMCB**, **BEMCB**, and mixed amine-50 fuel have good compatibility and stability. The stability of the fuel is crucial for ensuring the reliability of the fuel during storage and use. Good compatibility means that in practical applications, potential risks caused by material incompatibility can be reduced, thereby improving the safety and efficiency of the entire propulsion system.

#### 3.2.2. Physicochemical Properties

The physicochemical properties of propellant fuel are key determinants of fuel performance. Among these, the performance indicators that significantly affect propellant performance are density, freezing point, viscosity, and calorific value, which are crucial for the fuel’s performance in various application scenarios. Density affects the storage volume of the fuel, directly related to its energy density during application; the freezing point determines the fuel’s flowability at low temperatures, which is critical for its use under extreme temperature conditions; viscosity is related to the fuel’s lubricity and flowability within the engine, affecting the engine’s operational efficiency; and the calorific value is an important indicator for measuring the fuel’s energy release capacity, determining the fuel’s combustion efficiency. Based on this, relevant tests were conducted on the three samples listed in Table 1, and the test results are shown in Table 2.

Based on the data in the table, it can be seen that compared to the base fuel A, the compounded fuels B and C both have densities above 0.88 and their freezing points are both below −55 **°**C. The volumetric heat values have increased, with compounded fuel B at 36.36 kJ·cm^−3^ and compounded fuel C at 36.43 kJ·cm^−3^. In terms of viscosity at 20 **°**C, compounded fuels B and C are slightly higher than base fuel A. The viscosity of the compounded fuels is similar to that of hydrocarbon fuels like RG-1; therefore, they can be used as propellant fuels.

Based on the data in the table, it is evident that compounded fuels B and C show certain advantages over base fuel A in key performance parameters. Specifically, the densities of compounded fuels B and C are both above 0.88 g·cm^−3^, indicating their higher energy density, which allows for the storage of more energy in a limited space. For propellants, this is one of the important factors in enhancing engine performance. Meanwhile, the freezing points are both below −55 **°**C, meaning that in extreme low-temperature environments, the fuel can still maintain good flowability and will not freeze, affecting the normal startup and operation of the engine.

In terms of energy characteristics, the volumetric heat values of the compounded fuels have increased, with compounded fuel B at 36.36 kJ·cm^−3^ and compounded fuel C at 36.43 kJ·cm^−3^. Compared to base fuel A, this improvement means that under the same volume, the compounded fuels can provide more energy, which for space-limited propulsion systems, can effectively enhance their thrust and endurance capabilities.

In terms of viscosity at 20 °C, compounded fuels B and C are slightly higher than base fuel A, with viscosity similar to that of hydrocarbon fuel RG-1. The viscosity characteristics of compounded fuels B and C allow them to maintain higher energy density while still meeting the requirements for flowability and atomization performance during use as propellants; therefore, they can be used as propellant fuels.

#### 3.2.3. Thermal Stability Analysis

**Figure 6:**
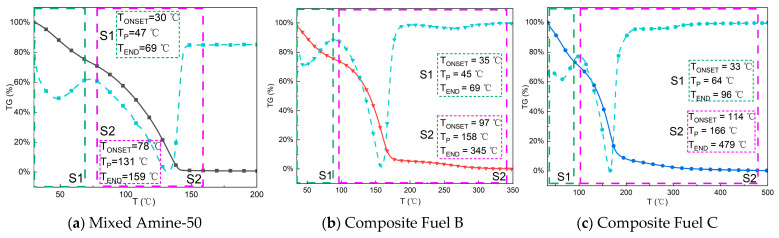
Thermo-gravimetric curves of Mixed Amine-50 and composite fuels. (Curves in black, red, and blue represent the thermogravimetric (TG) profiles of Mixed Amine-50, Composite Fuel B, and Composite Fuel C, respectively. Cyan curves correspond to their derivative thermogravimetric (DTG) profiles. Dashed boxes, color-coded by the thermal decomposition stage, delineate temperature ranges for distinct degradation processes.

To investigate the thermal stability changes of the compounded liquid propellants, TG analysis was performed on the base fuel A, compounded fuel B, and compounded fuel C using a thermogravimetric analyzer, and their TG-DTG curves were obtained, as shown in Figure 6.

The TG curves shown in Figure 6 indicate that the base fuel A undergoes significant mass loss in the temperature range of 30 °C to 69 °C, reflecting its high volatility. Within this temperature range, the mass loss of the base fuel A is mainly related to the evaporation of its volatile components, suggesting that the fuel has strong volatility at low temperatures and may easily lose some of its effective components during storage and use. In comparison, compounded fuel B also experiences mass loss in the temperature range of 35 °C to 69 °C, but its mass loss range is notably extended to 30 °C to 350 °C, exhibiting a broader thermal decomposition temperature range, especially at higher temperatures. This phenomenon indicates that the compounding of base fuel A with **BHMCB** (possibly an additive or stabilizer) significantly enhances the fuel’s thermal stability. The extension of the thermal decomposition temperature range of compounded fuel B suggests that the introduction of **BHMCB** effectively improves the fuel’s high-temperature decomposition tolerance, slowing down the volatilization and decomposition process at high temperatures.

Further analysis of compounded fuel C shows that its TG curve indicates a significant reduction in volatility in the temperature range of 35 °C to 90 °C. This change suggests that compounded fuel C has lower volatility at low temperatures, thereby reducing the loss of volatile substances. After the volatilization phase, the weight loss range of compounded fuel C is significantly expanded until 479 °C, indicating its good thermal stability and ability to maintain strong decomposition stability at higher temperatures. During this process, **BEMCB** (another additive or stabilizer) plays an important role in the thermal stability of the fuel. The introduction of **BEMCB** enables compounded fuel C to have stronger tolerance and decomposition stability at high temperatures, further enhancing the safety and reliability of the fuel under high-temperature conditions.

According to the analysis results of the differential thermogravimetric (DTG) curves, the thermal decomposition rates of each sample can be further discussed. After the volatilization phase, the maximum decomposition rate peak of base fuel A appears at 131 °C, indicating a relatively rapid pyrolysis process, which may lead to a significant loss of effective energy in a short time. Compared to base fuel A, the maximum decomposition rate peak temperature of compounded fuel B is increased to 158 °C, while compounded fuel C exhibits even better thermal stability, with a maximum decomposition rate peak temperature of 166 °C, further proving the role of **BHMCB** and **BEMCB** in enhancing fuel thermal stability. Among them, **BEMCB** has the most significant effect on improving the thermal stability of compounded fuel C, which is of great importance for the safe use of liquid propellants. In practical applications of liquid propellants, maintaining stable thermal performance at higher temperatures can not only effectively prevent premature fuel decomposition but also improve combustion efficiency, thereby ensuring the stability and reliability of the propellant.

In summary, based on the analysis of TG and DTG curves, compounded fuels B and C show significant improvements in volatility and thermal stability. Particularly, the introduction of **BEMCB** plays a key role in enhancing fuel thermal stability, corresponding to the previous thermal analysis results. This finding has important engineering significance for the design and safety of liquid propellants.

### 3.3. Theoretical Calculation of I_sp_

**Figure 7:**
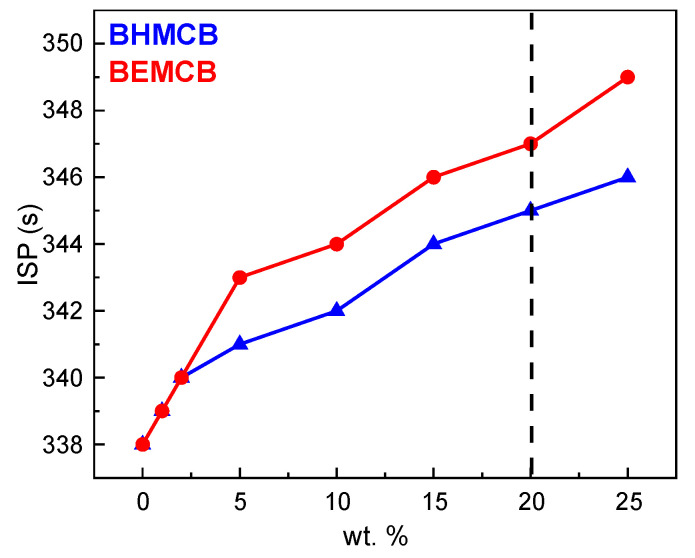
Effect of OCB, **BHMCB**, and **BEMCB** contents on systematic *I_sp_* performance.

To investigate the impact of the modified *o*-carborane modifier on the specific impulse performance of liquid propellants, the theoretical specific impulses of both systems with added fillers were calculated. The simulations were conducted using the NASA-CEA software (https://cearun.grc.nasa.gov, 6 March 2024), with the following specific calculation conditions: combustion chamber pressure of 68.02 atm, expansion ratio of 70, oxidizer as fuming nitric acid, base fuel as mixed amine-50, and an oxygen/fuel ratio of 4.5. The calculated vacuum equilibrium flow theoretical specific impulse for the base fuel A was 338 s. To further confirm the impact of the *o*-carborane modifier on specific impulse performance, theoretical vacuum equilibrium flow-specific impulse calculations were performed based on the system. The results are shown in Figure 7, whereby the *o*-carborane modifier still has a good effect on improving the specific impulse performance of the mixed amine-50 liquid propellant system, with little difference in the theoretical specific impulse performance compared to the original *o*-carborane, and the system’s theoretical vacuum equilibrium flow specific impulse is above 340 s.

To further illustrate the impact of fillers on the overall density-specific impulse performance, the density-specific impulses of the base fuel and compounded fuels were calculated, with the results shown in Table 3. According to the results in the table, calculated based on the configuration ratios in Section 3.2.1, the theoretical vacuum equilibrium flow specific impulse of the compounded fuels can be above 344 s, indicating that the addition of fillers has an enhancing effect on the fuel’s theoretical specific impulse performance. Additionally, the theoretical density-specific impulse calculation results show that the addition of modifiers has a significant effect on improving the overall density-specific impulse performance, with **BEMCB** having a better enhancing effect than **BHMCB**. Overall, the introduction of **BEMCB** not only increases the fuel’s density but also significantly enhances its specific impulse performance.

The density-specific impulse (D-Isp), a critical parameter for evaluating the comprehensive performance of propellants, is defined as the product of theoretical specific impulse (Isp) and fuel density (D-Isp = Isp × ρ). This metric reflects the total impulse generated per unit volume of fuel, directly correlating with the volumetric efficiency and energy output of propulsion systems. For spacecraft design, higher D-Isp values enable greater momentum delivery within fixed fuel volumes, a critical advantage for volume-constrained systems such as satellite orbit correction modules or high-maneuverability missiles. As shown in Table 3, the D-Isp of the **BEHMC**/mixed amine-50 composite propellant reaches 2.99 × 10^6^ N·s·m^−3^ at 20 % **BEHMC** loading, representing a 3.8% improvement over conventional mixed amine-50/red fuming nitric acid systems (D-Isp = 2.88 × 10^6^ N·s·m^−3^). This enhancement arises from two synergistic mechanisms: (1) Density Contribution: the high density of **BEHMC** (0.95 g·cm^−3^) significantly increases the overall fuel density (0.884 g·cm^−3^), (2) directly amplifying D-Isp Energy Release Optimization: the boron-rich cluster structure of **BEHMC** generates high-energy fragments during combustion. These fragments exhibit a volumetric calorific value of 36.36 kJ·cm^−3^, surpassing pure mixed amine-50 (35.97 kJ·cm^−3^) by 1.2%, thereby improving energy release efficiency per unit volume. The combined effects result in a D-Isp improvement exceeding the theoretical Isp gain (2.3%), demonstrating that **BEHMC** not only enhances the chemical-to-kinetic energy conversion efficiency but also strengthens the volumetric compactness of propulsion systems through density augmentation. From an engineering perspective, the D-Isp enhancement holds practical significance. For instance, in missions with fixed propellant tank volumes, higher D-Isp enables increased total impulse delivery, extending operational range or improving maneuverability. This aligns with the growing demand for high-payload, high-agility spacecraft in modern aerospace applications.

The calculation results indicate that introducing cage-like boranes into the liquid propellant system can significantly enhance its specific impulse performance, mainly attributed to their unique physicochemical properties. Cage-like boranes are a class of compounds with complex molecular structures composed of boron and hydrogen atoms, featuring a highly symmetrical molecular skeleton. In these molecules, the B-H chemical bonds are a key energy source, possessing high chemical energy density, which can rapidly release a large amount of energy during combustion, thereby significantly enhancing the propellant’s specific impulse. Furthermore, the electron-deficient radical fragments resulting from the breakdown of boranes can effectively enhance the redox reactions, promoting gasification reactions and increasing the molecular weight of gases per unit time. At the same time, cage-like boranes exhibit excellent thermal stability, with their unique molecular structure enabling them to maintain high thermal stability under high-temperature conditions, preventing premature decomposition during propellant use. This characteristic ensures that during rocket engine ignition and combustion, the propellant can efficiently and stably release a large amount of chemical energy, thereby maintaining efficient thrust output over a longer period. Particularly, typical cage-like boranes such as B_12_H_12_^2−^, B_10_H_10_^2−^, and B_6_H_7_^−^ have general thermal decomposition temperatures of around 200 °C, which gives them strong decomposition tolerance within conventional combustion temperature ranges, providing stable and efficient energy release [33]. Additionally, the high-density characteristic of cage-like boranes also plays an important role in enhancing specific impulse performance. Compared to traditional liquid fuels, cage-like boranes have a compact molecular structure and higher mass density, enabling them to store more energy within the same volume. Due to their higher density, propellants can store more fuel within the same volume, further enhancing specific impulse performance. This characteristic not only helps to increase the fuel’s energy density but also provides more optimization space for the design of liquid propellants, allowing for the storage of more energy within a limited volume, thereby enhancing the energy release efficiency during combustion.

## 4. Conclusions

This study has developed two new types of *o*-carborane derivatives, bis(hydroxymethyl)-*o*-carborane (**BHMCB**) and bis(2-ethylhexyl methacrylate)-*o*-carborane (**BEMCB**), both of which possess good stability, density, and energy properties. Among them, **BEMCB**, as a liquid cluster, has a broader application prospect compared to solid clusters. **BEMCB** has achieved the compounding of *o*-carborane cluster materials with mixed amine-50, resulting in a cluster liquid propellant with a density of 0.884 g·cm^−3^, a freezing point as low as −55 °C, a mass heat value as high as 41.13 kJ·g^−1^, a volumetric heat value as high as 36.36 kJ·cm^−3^, a theoretical specific impulse as high as 347 s, and a theoretical density-specific impulse as high as 2.99 × 10^6^ N·s·m^−3^, while also possessing good thermal stability, ensuring operational safety. Overall, the introduction of cage-like borane liquid clusters into liquid propellants, through their characteristics of high energy density, high thermal stability, and high density, has significantly improved the specific impulse of liquid propellants, making them perform exceptionally well in liquid propellants and offering broad application prospects, thereby holding important value in aerospace and military applications. These characteristics not only enhance the performance of propellants but also provide new ideas and methods for the design and application of liquid propellants.

## Figures and Tables

**Table 1:** Components and ratios of composite fuels.

Fuel	Components		wt. %
A	Mixed Amine-50	-	0
B		**BHMCB**	20
C		**BEMCB**	20

**Table 2:** Physicochemical properties of composite fuels.

Fuel	*ρ* (20 °C)/g·cm^−3^	FP/°C	μ/mPa·s	Q_v/_kJ·cm^−3^
A	0.855	<−55	1.02	35.97
B	0.884	<−55	2.26	36.36
C	0.884	<−55	2.32	36.43

**Table 3:** Theoretical calculations of Isp.

Fuel	ρ (20 °C)/g·cm^−3^	Isp/s	D-Isp/N·s·m^−3^
A	0.855	338	2.88 × 10^6^
B	0.884	344	2.96 × 10^6^
C	0.884	347	2.99 × 10^6^

## Data Availability

The original contributions presented in this study are included in the article. Further inquiries can be directed to the corresponding authors.
